# Novel H1N1 influenza infection in intensive care unit

**DOI:** 10.4103/0972-5229.78232

**Published:** 2011

**Authors:** Viroj Wiwanitkit

**Affiliations:** Wiwanitkit House, Bangkhae, Bangkok, Thailand

Dear Editor,

I read the recent report on novel H1N1 influenza infection by Chacko *et al*.,[1] with great interest. Chacko *et al*. concluded, “2009 H1N1 infection caused severe disease in relatively young patients without significant co-morbidities, characterized by severe hypoxemia and the requirement for prolonged mechanical ventilation” and “extra-pulmonary organ failure included circulatory and renal failure”.[1] The data from this work are similar to a recent report from Spain.[2] I would like to add some discussions on this work. First, there is no doubt that the novel H1N1 influenza can cause severe disease in healthy subjects. However, based on this work, it might not be possible to note that all patients have no co-morbidities since there are no data on health status of all cases prior to the present illness. The cause of detected prolonged mechanical ventilation should be discussed. The presented data that the affected cases had to be on ventilator for a week or more is not different from the cases with classical H1N1 influenza virus infection.[3] This might be due to no significant difference in pathogenesis between classical and novel H1N1 influenza infections. Finally, the extrapulmonary organ failure is important. Although it is not common, it is detectable.[4] However, based on personal experience, it can be said that the heart failure is extremely rare.[5]
